# Telerehabilitation After Total Knee Arthroplasty: A Narrative Review of Its Effectiveness, Safety, and Access in the Post-COVID Era

**DOI:** 10.7759/cureus.94102

**Published:** 2025-10-08

**Authors:** Samuelson E Osifo, Mutaleeb A Shobode

**Affiliations:** 1 Orthopaedic Surgery, University of Pittsburgh, Pittsburgh, USA; 2 Orthopaedic Surgery, Nationwide Children’s Hospital, Columbus, USA

**Keywords:** digital health equity, efficacy, knee arthroplasty, knee rehabilitation, osteoarthritis knee, patient outcomes, primary knee replacement, rehabilitation, telemedicine in orthopedics, telerehabilitation

## Abstract

Osteoarthritis (OA) is a leading cause of disability worldwide, affecting primarily older adults. With rising rates of obesity and population aging, the global burden of OA is expected to grow substantially. Total knee arthroplasty (TKA) remains the definitive treatment for end-stage knee OA. However, the growing demand for postoperative rehabilitation has intensified the strain on the physical therapy (PT) workforce. The COVID-19 pandemic accelerated the adoption of telerehabilitation, but evidence regarding its effectiveness, safety, cost-efficiency, and equity after TKA remains scattered.

A narrative review of English-language, full-text articles published between January 2018 and May 2025 was performed. PubMed, MEDLINE, and Google Scholar were searched using terms including “telerehabilitation”, “virtual physical therapy”, and “total knee arthroplasty”. Eligible studies were randomized controlled trials (RCTs), cohort studies, case series (n ≥ 10), or systematic reviews/meta-analyses that compared telerehabilitation with conventional in-person rehabilitation after TKA. Outcomes included functional metrics, e.g., Western Ontario and McMaster Universities Osteoarthritis Index (WOMAC), Oxford Knee Score (OKS), Knee Injury and Osteoarthritis Outcome Score (KOOS), and Knee Society Score (KSS), pain (visual analog scale, VAS), patient satisfaction, adherence, cost/utilization data, and equity indicators.

Eight studies (six RCTs, two meta-analyses; n ≈ 2,070) met the inclusion criteria. Interventions included AI-driven apps, video-based therapy, and wearable sensors. Most studies found telerehabilitation to be non-inferior to conventional PT in improving pain, range of motion (ROM), and function. Meta-analyses showed comparable gains in KOOS, ROM, and VAS scores. Cost-effectiveness was favorable in bundled care models, though technology costs were inconsistently reported. No increased adverse events were observed. Digital literacy and broadband access emerged as equity challenges.

Telerehabilitation is a safe, effective adjunct to in-person PT following TKA, with potential to expand access and reduce system strain. Hybrid models may offer the most sustainable path forward.

## Introduction and background

Osteoarthritis (OA) is a leading cause of disability worldwide, significantly impacting quality of life and mobility, particularly among older adults [1]. In the United States, an estimated 32.5 million adults are affected by OA. Globally, about 595 million people (≈7.6% of the population) were living with OA in 2020, and this figure continues to rise [2]. Driven by demographic aging, rising obesity rates, and sedentary lifestyles, the burden of OA is projected to increase substantially in the coming decades. By 2050, the number of persons living with OA is projected to reach one billion worldwide [2,3].

The knee is the most affected joint, accounting for most symptomatic cases and surgical interventions [3]. For patients with end-stage knee OA refractory to conservative management, total knee arthroplasty (TKA) is the standard of care. In the United States, more than 700,000 TKA procedures are performed annually [4], and this number is projected to continue to rise. Postoperative rehabilitation, particularly structured physical therapy (PT) and early ambulation, plays a pivotal role in functional recovery and long-term outcomes [5].

The growing demand for postoperative function recovery places increasing strain on rehabilitation services [6]. The U.S. Bureau of Labor Statistics has projected a 15-20% increase in demand for physical therapists between 2022 and 2032, outpacing the growth of the workforce [7]. This supply-demand mismatch underscores the need for scalable and accessible rehabilitation alternatives.

Telerehabilitation (telerehab), the remote delivery of PT services via digital platforms, has emerged as a promising adjunct or alternative to traditional in-person therapy [8-10]. It gained significant traction during the COVID-19 pandemic, which accelerated the adoption of telehealth modalities across healthcare. Advocates highlight its potential to improve access, reduce healthcare system burden, and maintain continuity of care in resource-limited settings [9,10]. Despite the growing use of telerehabilitation, evidence regarding its efficacy, safety, cost-effectiveness, and patient satisfaction is fragmented, often derived from heterogeneous studies with varying methodologies, technologies, and populations [8-11].

To date, there has been no unified synthesis focused exclusively on post-TKA telerehabilitation outcomes. This review addresses that gap by critically evaluating and integrating current evidence, identifying consistent findings, limitations, and future directions. By focusing specifically on telerehabilitation following TKA, we aim to provide a focused analysis to guide clinicians, inform policy, and support innovation in remote rehabilitation strategies.

## Review

Methods

We performed a comprehensive literature search of PubMed, MEDLINE, and Google Scholar (last update June 2025) using terms for telerehabilitation (tele-physical therapy (tele-PT), virtual physical therapy (virtual PT), remote rehabilitation) combined with “total knee arthroplasty (TKA).” Inclusion criteria were human studies in English (randomized controlled trials (RCTs), cohort studies, case series with ≥10 patients, and meta-analyses) comparing telerehabilitation to in-person rehabilitation after TKA. Outcomes had to include at least one of the following: functional scores, including Western Ontario and McMaster Universities Osteoarthritis Index (WOMAC), Knee Injury and Osteoarthritis Outcome Score (KOOS), and Oxford Knee Score (OKS), pain (visual analog scale (VAS) and numeric rating scale (NRS)), activity measures (e.g., step count), patient satisfaction/adherence, or economic/access metrics. We excluded non-TKA studies, case reports, and non-English reports.

Results

Study Selection and Characteristics

Eight studies met the final eligibility criteria, including four from the USA, one from Canada, one from Spain, and two from China; six RCTs and two systematic reviews/meta-analyses. Sample sizes ranged from small pilot trials (n≈25) to pooled meta-analyses with up to 1,944 participants. Table 1 lists the characteristics of the selected studies evaluating telerehabilitation after TKA.

**Table 1:** Characteristics of included studies evaluating telerehabilitation after TKA. Studies are organized by author, year, and country, with design, sample size, intervention, comparator, and follow-up schedule summarized. Follow-up times are expressed in weeks unless otherwise noted. Sample size is reported as randomized groups or completers when available. TKA: total knee arthroplasty; PT: physical therapy; VR: virtual reality; ERPM: enhanced recovery program model; RPT: remote physical therapy.

Study (author, year, country)	Design	Sample size (n)	Telerehabilitation intervention	Control group protocol	Follow-up (weeks)
Nuevo et al. (2024), Spain [12]	Prospective RCT	52 (26 vs. 26; 45 completers)	3 sessions/week AI-guided exercise + smart app	3 sessions/week home exercise program	Baseline; 2; 4
Bell et al. (2020), USA [13]	Pilot RCT	25 (13 vs. 12)	3 sessions/week via mobile app + clinician portal	Home exercise + single in-clinic visit	Baseline; 5; 10
Christiansen et al. (2024), USA [14]	RCT	92	1 session/week telehealth self-management + Fitbit sensor	Standard outpatient PT	Baseline; 8; 14; 38
Zhang et al. (2023), China [15]	Systematic review & meta-analysis	9 RCTs (n = 1,944)	Home-based supervised + self-directed exercise	Outpatient PT	1–52
Prvu Bettger et al. (2020), USA [16]	RCT (VERITAS)	306 (143 vs. 144 completers)	3 sessions/week avatar-led PT + 3D biometrics + remote PT	Traditional home/outpatient PT	12
Zhao et al. (2023), China [17]	RCT	100 (50 vs. 50)	3 sessions/week app-based rehab + wearable sensors	Written home rehab + outpatient visits	2; 6; 12
Gazendam et al. (2022), Canada [18]	Systematic review & meta-analysis	9 RCTs (n = 835)	VR-based telerehab	Traditional rehab	2; 6; 12; 24
Bradbury et al. (2024), USA [19]	RCT	197 randomized; 76 vs. 95 completers	6-week ERPM+RPT: 2 sessions/day remote PT + maintenance	6-week ERPM+OPT: 3 sessions/week + maintenance	Baseline; 6; 12; 52

Interventions varied, and app- or video-based exercise programs (some artificial intelligence (AI)-guided), web portals with clinician feedback, wearable sensors, or virtual-reality platforms were commonly used. All studies included a telerehabilitation arm compared to usual care, such as standard home exercises or outpatient physical therapy [12-19]. Follow-up periods ranged from a few weeks to one year postoperatively.

In most trials, telerehabilitation supplemented usual care rather than replacing it entirely. For example, a Spanish RCT delivered three weekly AI-guided sessions via a smart app versus a home exercise booklet [12], while the VERITAS (Virtual Exercise Rehabilitation In-Home Therapy Compared with Traditional Care After Total Knee Arthroplasty) trial by Prvu Bettger et al. combined remote avatar-led PT with biometric monitoring and three weekly sessions versus traditional home/outpatient PT [16]. Several studies also included at least one in-person assessment, typically at therapy initiation.

Efficacy and Outcome Measures

Primary endpoints included knee range of motion (ROM), pain (VAS/NRS), and functional scores (OKS, KOOS). Secondary outcomes included physical activity (daily step count), performance tests (timed up and go test (TUG), chair-stand, six-minute walk test (6MWT)), and patient-reported outcome measures (PROMs) (WOMAC, EuroQol 5-dimension (EQ-5D), Veterans RAND 12-Item Health Survey (VR-12)). Most trials reported no significant differences between telerehabilitation and conventional therapy, with small gains favoring telerehabilitation in specific metrics, such as KOOS or quadriceps strength [12,15,18]. Patient satisfaction remained high across modalities. Outcomes of included studies are summarized in Table 2.

**Table 2:** Outcomes and key findings of included studies on telerehabilitation after TKA. TKA: total knee arthroplasty; ROM: range of motion; TUG: timed up and go test; WOMAC: Western Ontario and McMaster Universities Osteoarthritis Index; EQ-5D-5L: EuroQol 5-dimension 5-level questionnaire; ADLS: activities of daily living scale; KOS: Knee Outcome Survey; aROM: active range of motion; NPRS: Numeric Pain Rating Scale; VR-12: Veterans RAND 12-Item Health Survey; 6MWT: six-minute walk test; LSA: Life-Space Assessment; KOOS: Knee injury and Osteoarthritis Outcome Score; OKS: Oxford Knee Score; KSS: Knee Society Score; SF-36: 36-Item Short Form Survey; 5×SST: five-times sit-to-stand test; SLST: single-leg stance test; VAS: visual analog scale; NRS: numeric rating scale; ERPM: enhanced recovery program model; RPT: remote physical therapy; OPT: outpatient physical therapy; PROMs: patient-reported outcome measures.

Study	Primary outcomes	Secondary outcomes	Equity/access	Key notes
Nuevo et al. (2024) [12]	ROM	Isometric strength; TUG; WOMAC; EQ-5D-5L	Not reported	↑ Adherence (p = 0.002); ↑ quad strength (p = 0.028); high usability; COVID limited enrollment
Bell et al. (2020) [13]	Cost-adjusted ADLS (KOS)	aROM; NPRS; VR-12; 6MWT; stair climb; TUG; balance test	Not reported	Value = ADLS change ÷ cost; small sample; high attrition; usability issues
Christiansen et al. (2024) [14]	Daily step count	LSA; 30-second chair stand; TUG; 6MWT; WOMAC; VR-12	Not reported	↑ Step count at 14 weeks, not sustained at 38 weeks; possible cross-contamination
Zhang et al. (2023) [15]	Pain; KOOS; ROM	EQ-5D-5L; OKS	Not reported	Hospital-based better for KOOS ≤14 weeks; telerehab better for ROM ≤14 weeks
Prvu Bettger et al. (2020) [16]	12-week healthcare costs	KOOS; rehospitalizations; falls; gait speed	Not reported	↓ Cost/readmissions; slight ↑ falls in the virtual group
Zhao et al. (2023) [17]	Knee ROM at 12 weeks	WOMAC; KSS; SF-36; 5×SST; SLST; satisfaction; costs; complications	Not reported	↑ ROM and functional performance; no difference in some PROMs
Gazendam et al. (2022) [18]	VAS pain	WOMAC; KOOS	Not reported	No pain difference; VR ↑ function at 12 & 24 weeks
Bradbury et al. (2024) [19]	NRS pain; knee ROM	VR-12; TUG; 4 m gait; satisfaction	Not reported	No clinical differences; RPT group avoided travel/time costs

Safety and Adherence

Telerehabilitation was well tolerated, with excellent compliance, including in older adults. Adverse events were rare. The VERITAS trial reported a slightly higher fall rate in virtual PT (19.4% vs. 14.6%), but this was not statistically significant [16]. Overall, safety outcomes, including readmissions and complications, were comparable between telerehabilitation and standard care.

Equity and Access

Most interventions required internet or smartphone access; one also included a simple wearable device [14]. Digital literacy, broadband limitations, and device access created barriers for rural or socioeconomically disadvantaged patients, leading to underrepresentation in trials and highlighting ongoing equity challenges [6,7].

Cost and Resource Utilization

Telerehabilitation consistently reduced in-person visits and travel burdens. The VERITAS trial showed median 12-week costs of $1,050 for virtual PT versus $2,805 for traditional PT (p < 0.001), with fewer readmissions [12]. Other systematic reviews reported similar trends [11]. Full economic evaluations remain limited, as device costs, software, and therapist time were not consistently included.

Discussion

Efficacy

Across randomized trials and meta-analyses, telerehabilitation following TKA generally yields clinical outcomes comparable to conventional therapy. Most studies found no significant differences between telerehabilitation (often home- or app-based programs, sometimes with remote monitoring) and standard in-person or home-based PT in primary endpoints like knee ROM, pain (VAS/NRS), or patient-reported function (e.g., KOOS and WOMAC) [12,16]. For example, Gazendam et al.’s meta-analysis of virtual reality (VR)-based post-TKA rehab reported no between-group differences in pain at early follow-up and found modestly greater functional scores with the VR intervention at 12 and 24 weeks [18]. Similarly, the VERITAS trial showed telerehabilitation (avatar-led exercises with remote PT oversight) to be noninferior to usual care on KOOS and objective strength and mobility measures, while dramatically lowering costs [16]. In essence, telerehabilitation appears to match the efficacy of traditional rehab for improving joint function and reducing pain in the short- to mid-term.

Notably, some studies reported specific benefits of telerehabilitation. A randomized trial using an AI-guided home-exercise app (ReHub®) found significantly higher exercise adherence and quadriceps strength in the telerehab group compared to standard home exercises [12]. Another trial combining telehealth coaching with activity trackers showed significantly higher daily step counts at 14 weeks (though this difference was not maintained at 38 weeks) [14]. Thus, while aggregate outcomes are similar, telerehab may offer advantages in engagement and targeted metrics (e.g., strength and physical activity) for some patients. Patient satisfaction has been high in both telerehab and in-person groups in reported trials (often aided by the usability of platforms), suggesting that patients generally accept remote therapy modalities.

Safety

Telerehabilitation has generally been safe and well-tolerated. Adverse events were rare and comparable between groups. For instance, the VERITAS trial noted a nonsignificant trend toward a higher fall rate in the telerehab arm (19.4% vs. 14.6%), but overall complications (falls, rehospitalizations) were similar across modalities [16]. Crucially, remote therapy did not increase readmission rates; in fact, VERITAS observed fewer rehospitalizations in the virtual PT group [16]. No trial reported serious safety issues attributable to remote care. Compliance with tele-exercises has generally been high; digital reminders, real-time feedback, and simpler home programs may bolster adherence [12]. In sum, telerehabilitation appears to be as safe as standard post-TKA rehab.

Equity and Access

Most telerehabilitation programs require internet access and a compatible device (smartphone, tablet, or computer), sometimes with wearable sensors. This introduces barriers for patients with limited technology access or digital literacy. Many studies implicitly screened for a minimum tech aptitude by excluding patients without broadband or smartphone access, so disadvantaged populations (e.g., very elderly, low socioeconomic status, or rural patients) are underrepresented in trials. Broadband disparities and digital literacy issues could limit the real-world reach of telerehabilitation. Addressing these challenges, for example, by providing devices, simplified user interfaces, or training, will be crucial.

Cost and Resource Utilization

A consistent advantage of telerehabilitation is reduced resource use. By enabling home-based therapy, telerehab cuts down on clinic visits and patient travel. The VERITAS trial provides a striking example: median healthcare costs over 12 weeks were about $1,050 per patient in the virtual PT arm vs. $2,805 in the usual-care arm [16]. This cost reduction reflects fewer PT clinic visits, less patient travel time, and fewer rehospitalizations in the telerehab group [16]. Systematic reviews of telehealth note similar trends; virtual programs often save costs even after accounting for devices and platform fees. However, full economic analyses are still sparse. Many studies report only direct therapy costs or travel savings; few incorporate device and infrastructure costs or therapist time for remote monitoring. Longer-term cost-effectiveness (e.g., over a year or in bundled payment models) remains to be established.

Hybrid Models and Prehabilitation

Nearly all current programs implement telerehabilitation as an adjunct or modification of usual care, not a wholesale replacement. For example, many trials include one in-person evaluation or integrate remote sessions into an overall outpatient rehab plan. The likely reason is to balance remote convenience with patient safety and personalized oversight. Hybrid models allow initial in-clinic instruction or assessment, followed by home-based telerehab. One underexplored opportunity is prehabilitation, i.e., using telerehab platforms before surgery to educate and strengthen patients. Preliminary data are promising. In one study, patients who used a tele-prehabilitation program had significantly shorter postoperative hospital stays (2.0 vs. 2.7 days) and were more often discharged home rather than to skilled nursing [20]. These early results suggest pre-surgery telerehab could improve outcomes and reduce costs, but large trials are needed.

Several factors limit current telerehabilitation programs. Technologies (apps, wearables, VR) vary widely in usability; complex interfaces or login procedures can frustrate older users. None of the trials formally reported on patients who declined enrolment due to tech barriers, but digital exclusion likely occurred. Ongoing design efforts should prioritize user-friendly platforms, larger text, voice commands, and initial onboarding support. Another gap is the lack of integration of emerging technologies: future systems could incorporate artificial intelligence for real-time form correction or progression algorithms, and advanced motion capture for objective kinematic monitoring. These could allow a single therapist to supervise many patients more effectively.

Research gaps remain. Long-term outcomes (beyond six to 12 months) for telerehab are unknown. Also, broader inclusion is needed: most studies have middle-class, tech-savvy participants, often omitting underserved groups. Future trials should target rural or lower-income populations to test scalability and equity of telerehabilitation interventions. Additionally, standardized reporting of adherence, satisfaction, and cost (including all components) would improve comparisons. Finally, the COVID-19 pandemic surge in telehealth highlighted both promise and pitfalls. Many clinicians and patients now expect tele-options, but robust protocols and training must be established for routine care outside crisis conditions.

## Conclusions

In summary, current evidence indicates that telerehabilitation after TKA produces clinical outcomes (pain relief, knee function, ROM) equivalent to standard in-person rehab. It offers the clear advantages of reduced travel and lower healthcare costs (as in the VERITAS trial) without compromising safety or patient satisfaction. To date, no high-quality study has found inferior results with telerehabilitation; indeed, some have shown improved exercise adherence or activity levels. The most effective use of telerehabilitation appears to be as part of a hybrid model, supplementing rather than entirely replacing traditional therapy, at least in the early postoperative period.

Success depends on patient engagement and access: clear instructions, technical support, and accommodation of individual needs (language, dexterity, vision) are essential. Innovations like tele-prehabilitation, AI-driven feedback, and wearable monitors hold promise for the future. Crucially, efforts must be made to ensure equitable access, providing devices or connectivity support to those who lack them. As the demand for post-arthroplasty rehab grows, integrating robust telerehabilitation pathways into standard care could help meet this need without sacrificing quality or widening disparities. Large, long-term trials and comprehensive cost-effectiveness studies are the next steps to confirm these findings across diverse populations and healthcare systems.
